# Rethinking variety testing to recognise mixing ability: bridging breeding, policy, and practice in diversified agriculture

**DOI:** 10.3389/fpls.2026.1770790

**Published:** 2026-03-18

**Authors:** Pierre Hohmann, Sebastian Kussmann, Christian Schöb, Diego Rubiales, Paolo Annicchiarico

**Affiliations:** 1 Department of Biology, Healthcare and the Environment, Faculty of Pharmacy and Food Sciences, Universitat de Barcelona, Barcelona, Spain; 2 Department of Environmental Systems Science, Institute of Agricultural Sciences, ETH Zürich, Zürich, Switzerland; 3 Grain Legume Breeding, Getreidezüchtung Peter Kunz (GZPK), Feldbach, Switzerland; 4 Instituto de Investigación en Cambio Global, Universidad Rey Juan Carlos, Móstoles, Spain; 5 Institute for Sustainable Agriculture, Consejo Superior de Investigaciones Científicas (CSIC), Córdoba, Spain; 6 Research Center for Animal Production and Aquaculture, Council for Agricultural Research and Economics (CREA), Lodi, Italy

**Keywords:** agroecology, common agricultural policy, intercropping, plant breeding, variety registration, variety testing

## Abstract

Agricultural diversification through species mixtures offers proven ecological and agronomic benefits, from improved nutrient cycling and yield stability to enhanced resilience against pests, diseases, and climatic stresses. Yet, the widespread uptake of these systems remains constrained by a fundamental gap: the absence of information on variety suitability for intercropping. Under current legislation, variety testing frameworks are designed for pure stand cultivation, thereby discouraging breeders from selecting *within* and *for* species mixtures, let alone registering mixture-adapted varieties. This Perspective article examines how current principles, policies, and procedures underpinning variety testing constrain the development and uptake of crop varieties suited for species mixtures. We synthesise evidence suggesting that integrating mixing ability into variety testing and registration could better align breeding incentives with farmer needs. Building on insights from a EU CAP Network Focus Group and long-standing forage and cereal-legume research, we outline conceptual and operational pathways to enable adaptation. A framework for action identifies key leverage points across five levels: breeding, regulation, farm networks, data infrastructures, and policy. Breeding must shift from isolated to interaction-based variety evaluation and selection, supported by traits and genomic predictors relevant for intercropping. When appropriate, official testing should add mixture sub-trials and recognise mixing ability as a sustainability trait, while linking practitioner networks and open data systems. Policy incentives and eco-labelling can help make diversity-oriented breeding economically viable. Rethinking breeding and variety testing in this way is pivotal for aligning breeding innovation, agricultural policy, and on-farm practice with the goals of resilient, diversified agroecosystems.

## 1 Introduction: diversification needs adapted varieties

Diversity in crop production through species mixtures, also referred to as crop associations, intercropping or mixed cropping, has been recognised as a cornerstone of sustainable farming systems. Species mixtures often outperform pure stands in resource-use efficiency, yield stability, and resistance to biotic and abiotic stresses (Kiær et al., 2009; Bedoussac et al., 2015; Justes et al., 2021). However, despite mounting evidence and renewed political interest in diversification under the European Green Deal and Farm to Fork strategies, adoption of species mixtures in the EU remains limited. Besides technical challenges, one key bottleneck lies in the absence of varietal information and breeding objectives adapted to mixtures (Annicchiarico et al., 2019; Lammerts van Bueren et al., 2011; Rubiales et al., 2023).

Modern crop varieties have almost exclusively been bred and tested in single-variety stand environments. Their selection environments, trait targets, and registration tests are all optimised for such pure stand conditions. Consequently, varieties that would perform well in mixtures, owing to traits such as balanced competitiveness (i.e. efficient resource acquisition without disproportionate suppression of companion species), complementary rooting patterns, or stress amelioration, are rarely identified, recognised, or rewarded. Farmers who wish to introduce species mixtures must rely on trial and error, anecdotal recommendations, or small local initiatives, rather than on transparent, standardised variety information.

The EU CAP Network Focus Group “Crop Associations, including milpa and protein crops” explicitly highlighted this issue, emphasising that the success of crop diversification depends on breeding and testing systems that can capture variety performance in mixtures. The focus group’s mini paper on cultivar testing (Hohmann et al., 2023) argued that breeders face both economic and regulatory disincentives to select for mixing ability, i.e. the capacity of a crop variety to perform well when grown with other plant species. Here, we extend that argument by calling for a systemic adaptation of variety testing at the interface of breeding, regulation, and practice, to make it fit for diversified agriculture globally, while grounded in the European policy context. Rather than advocating a single regulatory reform, this Perspective article integrates insights from breeding research, European policy initiatives, and practitioner experience to outline realistic pathways for adapting variety testing to diversified agriculture.

## 2 Rethinking variety testing: from pure stands to mixture performance

Variety testing was established in the mid-20th century to ensure that new varieties demonstrated measurable improvements over existing ones in yield, quality, and stability, while remaining identifiable through distinct phenotypic characteristics. The resulting frameworks of Distinctness, Uniformity, and Stability (DUS) and Value for Cultivation and Use (VCU) trials reflected an era in which genetic uniformity and pure stand productivity had become predominant in variety development. Today’s sustainability goals, however, require us to value adaptability and complementarity alongside productivity. In diversified farming systems, performance depends not solely on a variety’s individual vigour but on its interactions with other species and with the environment. Neglecting this dimension creates a structural bias: varieties that thrive in mixtures may be rejected because they appear suboptimal in pure stand trials. Conversely, varieties highly competitive in pure stands may suppress partner species when mixed, undermining the total system yield.

Breeding research has shown that selection for intercropping is feasible and effective (e.g. Abou-Khater et al., 2024; Annicchiarico and Proietti, 2010; Haug et al., 2023). Decades of work on legume-grass forage systems and, more recently, grain cereal-legume intercrops have demonstrated that variety performances across pure stand and mixed cropping may be largely inconsistent, especially for the species that tends to be outcompeted under the adopted intercropping conditions with regard to nitrogen fertilisation level, sowing densities, vigour of the associated species and variety, etc (Annicchiarico et al., 2019; Brooker et al., 2024). To be effective, variety testing for adaptation to intercropping ought to focus not only on yield competition dynamics but also on functional complementarity. It requires both methodological innovation (new experimental designs and indices) and institutional adaptation (regulatory recognition of system performance).

## 3 Current challenges in integrating mixing ability into testing systems

### 3.1 The combinatorial complexity of species mixtures

The first obstacle to obtain variety suitability information for mixed cropping is logistical: there is an indefinite number of possible species combinations and variety combinations whenever specific mixing ability (SMA; i.e. when performance depends on the specific genotype of the mixture partner) is not negligible. Each new cropping partner or management condition multiplies experimental space and efforts. Testing all combinations is impossible. However, evidence from grass-perennial legume and cereal-annual legume intercrops indicates that general mixing ability (GMA) - reflecting genotype performance averaged across partners genotypes - explains a substantially larger share of performance variation than SMA (Annicchiarico et al., 2019; Gallais, 1970; Hamblin et al., 1976; Haug et al., 2023). Besides, SMA effects may largely depend on the vigour and competitive ability of the associated species and variety (Annicchiarico and Piano, 1994). This means that using a few, carefully chosen testers can capture most variation in performance, making mixture evaluation manageable within existing testing budgets. This tester approach relies on above observations and applies whenever one or a small number of testers captures most relevant interaction variance. Applicants seeking registration of a candidate variety could request that VCU testing be conducted in mixture with a specified companion species, to limit testing to the cultivation systems for which the cultivar is intended. Efficient designs, such as incomplete factorials and the use of standard testers, can further reduce the testing workload (Haug et al., 2021; Moutier et al., 2022). Nonetheless, variety testing organisations will need to balance representativeness with cost and throughput, possibly by embedding mixture sub-trials within existing networks. The breeding process could be further facilitated by modelling tools that simulate the outcome of the combination of different species traits for growth and yield development, reducing the need for extensive field testing (Weih et al., 2022).

### 3.2 Environmental and management interactions

Mixing ability is not fixed; it is shaped by Genotype x Environment x Management (GxExM) interactions. Studies across Europe have shown that the yield balance between partners in wheat-legume or pea-barley mixtures varies strongly with nitrogen inputs, sowing density, and site conditions (Moutier et al., 2022). The same has been reported for perennial forages, such as grass-white clover mixtures (Harris, 1987). Capturing these dynamics requires multi-environment testing representative of real farming contexts. Living-lab and on-farm networks offer a promising vehicle for this integration. While incorporating GxExM dimensions increases short-term testing costs, it also increases longer-term efficiencies by the release of varieties suited to diversified cropping systems, lowering farmer trial-and-error costs and increasing adoption stability. From a system perspective, these investments are likely to yield positive returns through improved yield stability and reduced input dependence.

### 3.3 Regulatory frameworks designed for pure stand cultivation

Perhaps the most entrenched barrier lies in the legal architecture of seed marketing. In all UPOV (Union for the Protection of New Varieties of Plants) contracting parties and in countries with sui generis plant variety protection systems, official DUS and VCU trials are mandatory for registration and commercialisation, and rely on pure stand tests. Cultivars bred for mixtures may thus fail VCU testing because of modest performance in pure stands even if they excel in mixtures (Chable et al., 2011). The proposed EU Seed Law revision introduces the notion of “sustainability traits” in testing, a potentially transformative step, but the operational definition of such traits remains vague (European Parliament, 2023). While the current definition of sustainability traits remains broad, ongoing legislative discussions suggest that operational clarification is likely as implementing acts and delegated regulations are developed. Recent EC Horizon 2020 projects such as *ReMIX*, *DIVERSify*, and *DiverIMPACTS* have delivered policy briefs on the importance of plant breeding as a structural bottleneck to diversification. These multi-actor projects converged in recommending that “mixing ability” be recognised as a sustainability trait within future DUS/VCU protocols.

### 3.4 Limited breeder incentives and market demand

The above multi-actor projects also suggested that breeders receive institutional incentives to include mixture performance in selection schemes. With no recognition of the agroecological value of mixing ability in formal variety testing, and with currently small areas of mixed stand cultivation, few breeding programmes invest in this direction. This creates a circular dependency: farmers do not adopt species mixtures because no adapted varieties exist, while breeders do not breed them because of a lack of official testing and markets. Although public funding has supported research on species mixtures for decades, these efforts have rarely translated into sustained incentives within official testing or market frameworks. As a result, mixture-oriented breeding remains largely project-bound rather than institutionally embedded. New collaborative breeding initiatives and inclusion of mixture trials in official testing could break this cycle by increasing visibility for producers and breeders engaging with diversity-oriented targets.

### 3.5 Fragmented information systems for farmers

Even where relevant varieties are available, reliable information on their mixture performance and on seed availability remains fragmented and difficult to access. Databases such as organicXseeds demonstrate how transparent, cross-border information systems can improve seed access (Lammerts van Bueren et al., 2011). Extending such models to include mixture suitability, linking official trial data, participatory networks, and private experiments, would empower farmers to make evidence-based decisions on variety combinations suited to their conditions.

## 4 Pathways towards integrating mixing ability: implications for breeding and variety testing

### 4.1 Using existing variety trials as entry points

Transforming variety testing does not necessarily require creating new infrastructures. Many public and private networks already evaluate varieties under contrasting environments and managements. A first step can be to embed mixture sub-trials into existing variety testing frameworks. For example, a set of candidate lines of a cereal crop species could be tested not only in pure stand but also in combination with a standard legume “tester” variety, if the applicant for variety registration request testing in mixture. However, most national testing authorities still lack legal flexibility to include mixture trials within official protocols, and results from such trials are often treated as “non-comparable” with pure-stand performance. Yet there are encouraging examples pointing to change: The tester approach has long been used for forage crops in Switzerland, where the competitive ability of clover and grass varieties are assessed in both pure stand and mixed stand, using a mixture of recommended varieties as a tester to estimate the competitive ability and mixture balance of candidate varieties (Hohmann, 2025). In cereal-legume systems, studies using standard legume testers have shown that a limited number of partners can reliably rank cereal genotypes for mixing ability across environments (Annicchiarico et al., 2019; Haug et al., 2023). In France, ARVALIS initiated pilot trials in which cereal-legume mixtures are evaluated alongside standard VCU tests, while in the UK, NIAB has explored similar setups using oat and pea. Such integration enables rapid accumulation of data on mixing ability without duplicating field networks or compromising standard VCU assessments.

### 4.2 Leveraging trait-based and genomic prediction

Because full mixture testing is resource-intensive, trait-based proxies and genomic prediction provide complementary routes. Traits such as plant height, early vigour, phenology, or root architecture often correlate with mixing ability in cereal-legume mixtures (e.g. Annicchiarico, 2003; Hauggaard-Nielsen and Jensen, 2001; Haug et al., 2023; Schöb et al., 2023). In particular, plant height and early vigour expresses high relative growth rate, which is a key trait to compete for light under moderately favourable growing conditions (Annicchiarico et al., 2019). Measuring these traits in pure stands allows preliminary ranking of candidate varieties before confirmatory mixture trials. Genomic prediction models trained on mixture-performance data can further accelerate selection for intercropping by estimating the expected general mixing ability of untested genotypes (Annicchiarico et al., 2025; Wolfe et al., 2021). Because of interactions of genotypes with pure stand or mixed stand conditions, genomic models trained on pure-stand data were less accurate than those trained on data collected in mixed stand to predict genotype performance in the latter conditions (Annicchiarico et al., 2025). The combination of physiological insight and statistical modelling could make mixture selection feasible within existing breeding budgets.

### 4.3 Participatory testing and living-lab networks

Living-lab and on-farm research networks offer unique opportunities to extend variety testing beyond research stations. They enable decentralised, multi-actor evaluation of variety performance in target management contexts (Hauggaard-Nielsen et al., 2021). Farmers contribute practical knowledge and management diversity, and scientists contribute design and analysis. Data from these networks could feed directly into official variety evaluations if harmonised protocols were adopted. The *DiverIMPACTS* and *ReMIX* projects have shown that coordinated farmer trials across countries can generate robust insights into GxExM interactions. Recent examples include the French DEPHY network and the German “Betriebsnetzwerk Ökolandbau”, which already collect mixture data at farm scale (de Witte, 2023; MASA – Ministère de l’Agriculture et de la Souveraineté Alimentaire, 2024). Recognising well-documented living-lab results as complementary evidence for variety registration could bridge the gap between participatory science and regulation. Yet, data quality and traceability remain major challenges: farmers rarely use uniform scoring scales or metadata standards, and statistical power can be low when management practices vary strongly. Data quality can be improved through simplified but standardised protocols, mandatory metadata (e.g. sowing density, fertilisation), and the inclusion of reference pure-stand plots for calibration. Metrics such as the Land Equivalent Ratio, which compares mixture yield to the summed yields of component pure stands, provide a robust, system-level benchmark that is compatible with heterogeneous on-farm data. Further, a European-wide harmonisation effort, perhaps under the SCAR-AKIS umbrella, could formalise data curation while keeping farmer participation central.

### 4.4 Digital infrastructures for data aggregation and decision support

A modernised variety testing system should include digital platforms that collate and disseminate mixture-performance data to better link and complement the formal registration process with more diverse post-registration, informal testing schemes. Examples such as *SeedLinked*, the *AgroDiversity Toolbox*, or the *CropMixer* decision-support tool illustrate how user-generated and research data can be integrated into open databases. A few countries such as Denmark (SortInfo), UK (AHDB Knowledge Library), and Spain (GENVCE) already publish multi-environment variety testing results in accessible online formats; an excellent foundation to which mixture data could be added. Developing a pan-European (and eventually global) portal that links official testing results, participatory data, and seed availability, would provide farmers and advisers with actionable information on mixture-adapted varieties. Standardised metadata and open-data policies, as developed in the *DIVERSify* project, will be essential to ensure interoperability and transparency. Current fragmentation of databases and limited willingness of breeding companies to share data still impede progress; clear data-governance rules and FAIR-data incentives will be required to unlock the full potential of digital infrastructures.

### 4.5 Policy and incentive frameworks

Policy can catalyse change. Recognition of mixing ability as a sustainability trait within EU VCU testing would provide an immediate incentive for breeders. Complementary incentives such as research grants, innovation awards, or temporary registration routes for varieties intended for mixtures would further de-risk investment. For example, the Austrian Agency for Health and Food Safety (AGES) already grants temporary marketing authorisation for “heterogeneous material” a mechanism that could be extended to mixture-adapted lines. Public procurement and eco-labelling schemes rewarding products from species mixtures could create market pull. National administrations could also encourage cross-border seed exchange and information sharing by extending platforms such as *organicXseeds* to all production systems. However, current legislation still treats mixtures as exceptions rather than a mainstream innovation pathway. Without coordinated operational changes to the EU Seed Marketing Directives and national variety registration rules, breeders remain disincentivised to select for mixing ability. Collective action between regulators, seed companies, and farmers will be necessary to align incentives and make diversity-oriented breeding economically viable.

## 5 A synthesis framework for integrating mixing ability into variety testing

This framework synthesises leverage points identified across the literature, policy initiatives, and practitioner networks, rather than proposing a prescriptive implementation sequence. Integrating mixing ability into variety testing requires coordinated progress across breeding, regulation, farm networks, data infrastructures, and policy. **Table 1** summarises leverage points at each level. At the breeding stage, selection must evolve from isolated performance towards interaction-based evaluation. Trait-based and genomic proxies can guide early screening, but selection and testing in species mixtures remain essential to validate ecological complementarity. For official variety testing, adding mixture sub-trials and recognising mixing ability as a sustainability trait would align registration criteria with agroecological goals without undermining existing DUS/VCU standards. Linking on-farm and participatory network datasets with official variety testing systems would expand environmental coverage and farmer engagement. Open digital infrastructures can support the integration of mixture data from breeders, researchers, and farmers, enabling transparent variety comparison and choice. Finally, policy incentives, such as temporary registration routes, funding of breeding for mixtures, and eco-labelling, can mainstream diversity-oriented breeding into an economically viable approach.

**Table 1:** Leverage points for integrating mixing ability into variety testing.

Level	Current limitation	Proposed innovation	Expected outcome
Breeding programmes	Selection and evaluation in pure stands only	Incorporate phenotypic and genomic prediction of mixing ability; use limited “tester” species in early stages	Efficient early selection of mixture-adapted lines
Official variety testing	DUS/VCU focus on pure-stand yield	Add mixture sub-trials with standard testers; recognise mixing ability as sustainability trait	Registration criteria reflecting performance in diversified systems
On-farm and participatory networks	Fragmented, non-standardised mixture trials	Harmonise protocols and share data with official systems	Broader environmental coverage and farmer engagement
Information and digital systems	Scattered data, limited access	Develop open-access databases and decision-support tools	Transparency and informed variety choice
Policy and market	Lack of incentives or recognition	Provide funding, temporary registration, eco-labelling	Increased breeder incentives and farmer uptake

## 6 Conclusions

Rethinking variety testing to include mixing ability represents more than a technical adjustment: it signals a cultural and institutional shift in how agricultural innovation is evaluated. The goal is not to replace pure stand testing but to complement it with system-level performance metrics that reflect modern sustainability objectives. This transformation will require cooperation across scientific disciplines and stakeholder groups. Breeders must collaborate with agronomists and ecologists to define measurable indicators of complementarity; testing authorities must adapt protocols without compromising rigour; farmers must be empowered as co-experimenters; and policymakers must ensure that regulations reward rather than penalise diversity. The emerging paradigm treats the field as an ecological community rather than a set of isolated genotypes. This vision aligns with conclusions from European multi-actor research initiatives, which translate scientific evidence on species mixtures into concrete policy recommendations for breeding and seed-testing reforms. By referencing these initiatives, the present paper calls for regulatory and operational innovation to accompany scientific advances.

International experience suggests that such integration is achievable. Examples like Switzerland’s forage crop testing system in mixture and various participatory breeding networks provide precedents. As digital infrastructures and data-science tools mature, the ability to synthesise multi-environment results will improve dramatically, enabling predictive, evidence-based decision-making for both breeders and farmers. Ultimately, recognising mixing ability within variety testing aligns production with agroecological principles: systems function best when components complement rather than compete excessively. Integrating this understanding into official frameworks would bridge the current divide between breeding science, agricultural policy, and practical farming. This integration is an essential step towards resilient and diversified agroecosystems.

Variety testing sits at the crossroads of science, regulation, and practice. Reforms will determine whether crop diversification remains a niche experiment or becomes a mainstream strategy for sustainable agriculture. By integrating mixing ability into testing and registration, we acknowledge the complex realities of diversified systems and give breeders, farmers, and policymakers the evidence needed to act. Well-designed adaptations in variety testing systems require only modest methodological adjustments, yet they may bring agricultural innovation into alignment with the ecological foundations on which it ultimately depends.

## Data Availability

The raw data supporting the conclusions of this article will be made available by the authors, without undue reservation.
